# 4D‐Flow MRI‐Derived Wall Shear Stress as a Non‐Invasive Biomarker for the Detection and Hemodynamic Characterization of Pulmonary Hypertension

**DOI:** 10.1002/pul2.70426

**Published:** 2026-10-19

**Authors:** Ben Statton, Chlöe Armour, Kai'en Leong, Jan Sedlacik, Alaine Berry, Ning Jin, Daniel Giese, Francesco Lo Guidice, Luke Howard, Deepa Gopalan

**Affiliations:** ^1^ Medical Research Council, Laboratory of Medical Sciences Imperial College London London UK; ^2^ Institute of Clinical Sciences Imperial College London London UK; ^3^ National Heart and Lung Institute Imperial College London London UK; ^4^ Department of Chemical Engineering Imperial College London London UK; ^5^ Department of Cardiology Royal Melbourne Hospital Melbourne Australia; ^6^ Siemens Healthineers Erlangen Germany; ^7^ National Pulmonary Hypertension Service Imperial College Healthcare NHS Trust London UK

**Keywords:** 4D flow cardiovascular magnetic resonance, computational fluid dynamics, pulmonary hypertension, wall shear stress

## Abstract

Invasive right heart catheterization (RHC) remains the reference standard for diagnosing pulmonary hypertension (PH), but there is a pressing need for non‐invasive techniques to assess pulmonary hemodynamics. This study evaluates 4D‐flow MRI‐derived wall shear stress (WSS) for PH detection using the updated 2022 hemodynamic criteria, and the prediction of right ventriculo‐pulmonary arterial (RV‐PA) uncoupling to assess disease severity. 3D WSS was measured in the main (MPA), right (RPA), and left (LPA) pulmonary arteries in a retrospective validation cohort (41 PH patients, 23 non‐PH) and then assessed in a prospective test cohort (23 PH patients, 17 healthy controls). For methodological validation, a patient‐specific computational fluid dynamics (CFD) was performed in a subset of 16 patients from the validation cohort. RV‐PA uncoupling was assessed on the cine MRI, using RV global longitudinal strain (GLS) < −15.3% and RVSV/RVESV < 1. Both 3D peak and time‐averaged WSS were significantly reduced in PH patients compared with healthy controls. Global WSS metrics demonstrated the strongest correlations with invasive hemodynamic parameters, including mPAP (*r* = −0.75, *p* < 0.001) and PVR (*r* = −0.76, *p* < 0.001). ROC curve analysis identified a peak WSS cutoff of < 0.30 Pa (AUC 0.98) for PH detection, a threshold achieving 90% sensitivity and 100% specificity in the test cohort. Furthermore, a peak WSS threshold of < 0.18 Pa demonstrated excellent discriminative ability for RV‐PA uncoupling. These findings support the potential integration of WSS analysis into routine CMR protocols as a non‐invasive complement to RHC, showing promise for both diagnosis and a longitudinal biomarker for monitoring PH.

## Graphical abstract

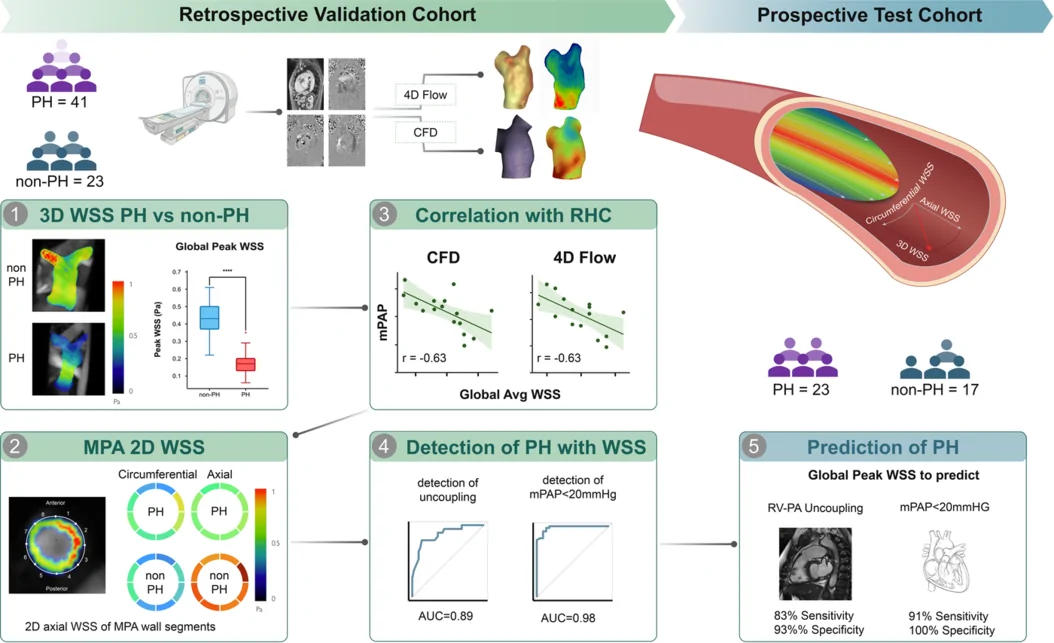

## 1 Introduction

Pulmonary hypertension (PH) is a progressive hemodynamic disorder characterized by pathological elevations in pulmonary arterial pressure and vascular resistance, ultimately leading to right ventricular (RV) failure [1]. The hemodynamic definition of PH was updated in 2022 to capture patients at earlier stages of the disease, now classifying PH with a lower mean pulmonary artery pressure (mPAP) of 20 mmHg at rest, and the addition of the mandatory criterion of pulmonary vascular resistance (PVR) > 2 Wood units (WU) [2].

Right heart catheterization (RHC) remains the clinical reference standard for the diagnosis and hemodynamic evaluation of PH [3]. However, due to its invasive nature, there is an emergent need for the development of robust, non‐invasive imaging techniques to comprehensively assess pulmonary hemodynamics and vasculature [4].

In recent years, cardiovascular magnetic resonance (CMR) imaging has emerged as the non‐invasive reference standard to assess cardiac function in PH, facilitating detection of early changes in RV function caused by elevated arterial pressure [5]. Alongside standard CMR, time‐resolved three‐dimensional phase‐contrast MRI with three‐directional velocity encoding (4D‐flow MRI) allows for the comprehensive evaluation of complex blood flow in the RV and proximal pulmonary arteries [6]. Previous 4D‐flow MRI studies have consistently demonstrated that patients with PH exhibit abnormal hemodynamics, including characteristic vortical flow in the main pulmonary artery (MPA) [7, 8] and reduced wall shear stress (WSS) in the proximal pulmonary arteries [9, 10, 11, 12] compared to healthy controls.

WSS is the primary mechanical drag force induced by blood flow along the endothelial surface of the vessel and serves as an important regulator of vascular health [13]. Abnormally low or disturbed WSS stimulates vascular remodeling, progressive vessel stiffening, and luminal dilation [14]. Consequently, quantifying WSS provides a valuable, non‐invasive window into the fluid‐tissue interactions that drive PH progression [15].

As pulmonary vascular resistance increases in PH, the resulting afterload severely disrupts right ventriculo‐pulmonary arterial (RV‐PA) coupling [16]. Once the right ventricle can no longer adapt to the increased pulmonary vascular load, RV‐PA uncoupling occurs, significantly increasing the risk of mortality [17]. In recent years, CMR‐derived RV global longitudinal strain (GLS) and volumetric approximation have been used as a surrogate non‐invasive marker of coupling, with a GLS of > −15.3 and RV Stroke Volume/RV End Systolic Volume ratio > 1.0 being indicative of preserved coupling [18, 19]. By acquiring comprehensive MPA flow hemodynamics with 4D‐flow and RV functional measures with cine imaging, an MRI examination offers a unique opportunity to correlate altered WSS directly with these markers of RV‐PA uncoupling.

In addition to the established differences in WSS between PH and healthy controls [20, 21], recent studies have demonstrated differences in peak and average WSS between pre‐ and post‐capillary PH [22], and improved WSS in the MPA after balloon pulmonary angioplasty in patients with chronic thromboembolic PH [23]. However, a universally cited limitation in the current literature is small sample size, which restricts statistical power and limits the translation of these results into diverse clinical settings where broader population representation is required. Furthermore, the studies that established reduced WSS in PH were conducted under the previous hemodynamic definition of PH, so may not have captured patients at earlier stages of the disease. These changes to the hemodynamic thresholds have the potential to decrease the observed differences in WSS between health and PH and to undermine the clinical applicability of previously reported results.

Despite the knowledge of complex vortical flow that may exert multidirectional shear forces on the MPA vessel wall in PH, few studies have investigated the separate axial and circumferential components of WSS [9]. Finally, due to the limited spatial and temporal resolution of 4D‐flow, true WSS magnitudes are often systematically underestimated [24, 25]. A recent study found that time‐averaged WSS from computational fluid dynamic (CFD) simulations was significantly higher than from 4D‐flow in PH patients [26]. The same authors found that the WSS spatially averaged over the surface of the MPA and the first bifurcation into the left pulmonary artery (LPA) and right pulmonary artery (RPA) demonstrated a strong correlation between the WSS and mPAP (*R* ^2^ = 0.84), albeit in a cohort of only seven patients [26].

To address these gaps, we present a larger 4D‐flow MRI study that seeks to establish a WSS cutoff value for detecting PH and then testing this in a separate cohort of patients. We hypothesize that spatially averaging WSS across the MPA, RPA, and LPA, following the approach of Armour et al., yields a more reliable metric that minimizes voxel‐level noise associated with the limited spatial resolution of 4D‑flow MRI and operator plane‐placement variability. However, recognizing that equal vessel weighting may mask regional hemodynamic heterogeneity, we also examine WSS separately in these three vessels between healthy controls and PH subtypes. We hope to characterize the alterations in WSS in the proximal pulmonary arteries and investigate the axial and circumferential components to better understand the changes in multidirectional flow. Furthermore, we sought to correlate these WSS metrics with invasive pressure measures and with CMR‐derived markers of RV‐PA uncoupling to evaluate WSS as a potential marker of early right ventricular maladaptation. Finally, to address the known limitation of relatively low spatial and temporal resolution of 4D‐flow MRI, we incorporate a high‐fidelity patient‐specific CFD sub‐analysis to validate and provide confidence in our 4D‐flow derived WSS values. To our knowledge, this current study represents the first investigation to use 4D‐flow WSS to discriminate between PH patients and the healthy population since the updated hemodynamic definitions of PH were established.

## 2 Methods

### 2.1 Subjects

To assess the ability of WSS to predict PH, two separate cohorts of patients and healthy volunteers were included in this study, the first “validation” cohort was retrospectively recruited from consecutive patients with known or suspected PH who had undergone CMR including 4D‐flow. Inclusion was restricted to adults (> 18 years) who had undergone CMR and RHC within 30 days. This validation cohort also included 23 healthy adult volunteers (> 18 years) with no history of cardiovascular disease who provided written informed consent. The results of the analysis in this validation cohort were then used to predict PH and RV‐PA uncoupling in a test cohort of prospectively recruited patients referred for CMR with suspected PH, who also underwent RHC during the same hospital visit and 17 healthy volunteers (Figure 1). Patients in this test cohort provided written informed consent and the inclusion criteria were the same as for the validation cohort.

**Figure 1:**
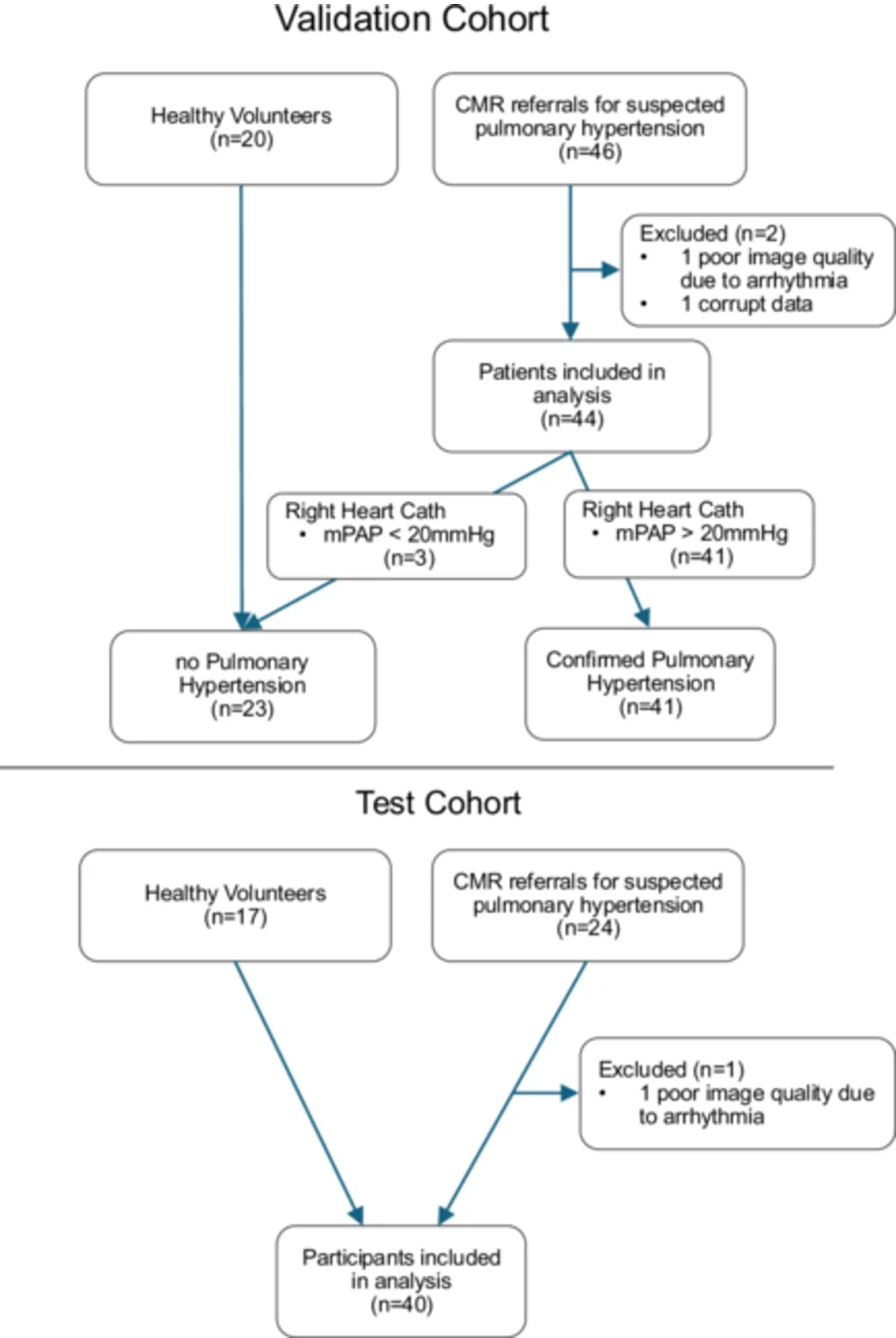
Patient flow. Patient and healthy volunteer flow diagram to validation cohort (upper panel) and test cohort (lower panel).

Regional Health Research Authority ethical approval was granted for both cohorts of this study, and the methods contributing to this work were carried out in accordance with the ethical standards of the relevant guidelines and regulations and with the Helsinki Declaration of 1975 and its subsequent amendments and clarifications.

### 2.2 MRI Acquisition

MR imaging was performed using a 1.5 T scanner (MAGNETOM Aera, Siemens Healthineers, Erlangen, Germany) and a 60‐channel cardiac coil. Each participant underwent a standard CMR protocol including retrospectively ECG‐gated long and short‐axis cines acquired during end‐expiratory breath‐hold with a balanced steady‐state free precession (bSSFP) sequence. Typical parameters for the bSSFP sequences were TR 2.8 ms, TE 1.18 ms, flip angle 59°, voxel size 1.5 × 1.5 × 8.0 mm^3^, bandwidth 947 Hz/pixel, and a Generalized Autocalibrating Partially Parallel Acquisition (GRAPPA) [27] factor of 2.

A prototype retrospectively ECG‐gated phase‐contrast 4D‐flow sequence was acquired with respiratory navigation employing a Respiratory Controlled Adaptive k‐space Reordering (ReCAR) technique to improve the navigator efficiency [28]. The 4D‐flow volume was acquired in the RVOT plane encompassing the RV outflow tract, MPA, and proximal portions of the LPA and RPA. The acquisition parameters followed consensus guidelines [29], TR 4.8 ms, TE 2.3 ms, flip angle 7°, bandwidth 947 Hz/pixel, GRAPPA 2, voxel size 2 × 2 × 2mm^3^, temporal resolution 43 ms, with 25 reconstructed cardiac phases. The appropriate velocity encoding (VEnc) was selected from a VEnc scout and ranged from 100 to 200 cm/s. The mean acquisition time ranged from 8 to 15 min depending on the patient's cardiac and respiratory rates. Subjects in both the validation and test cohort underwent this same MRI protocol.

### 2.3 MRI Analysis

Functional and volumetric assessment of the CMR cine images and analysis of the 4D‐flow data were performed in CVI42 (version 6.2.2, Circle Cardiovascular Imaging; Calgary, Canada). Volumetric analysis was performed following current guidelines [30], and biatrial and biventricular feature tracking (FT) strain was calculated as previously described [31]. For WSS analysis, images were pre‐processed for background error correction and phase unwrapping, then a 3D MRA generated from the phase contrast flow series was used to segment the pulmonary arteries using center line tracing. Planes were manually placed in the MPA, RPA, and LPA to measure systolic peak 3D WSS (WSS_peak_) and time‐averaged 3D WSS (WSS_avg_). Furthermore, composite metrics of global peak WSS (WSS_global_peak_) and global average WSS (WSS_global_avg)_ were obtained by calculating the arithmetic mean of all three analysis planes:

$${\mathrm{WSS}}_{\mathrm{global\_peak}}=\frac{{\mathrm{MPA}\;\mathrm{WSS}}_{\mathrm{peak}}+R{\mathrm{PA}\;\mathrm{WSS}}_{\mathrm{peak}}+{\mathrm{LPA}\;\mathrm{WSS}}_{\mathrm{peak}}}{3}$$

$${\mathrm{WSS}}_{\mathrm{global\_avg}}=\frac{{\mathrm{MPA}\;\mathrm{WSS}}_{\mathrm{avg}}+R{\mathrm{PA}\;\mathrm{WSS}}_{\mathrm{avg}}+{\mathrm{LPA}\;\mathrm{WSS}}_{\mathrm{avg}}}{3}$$

To evaluate their relative contributions to the 3D WSS described above, the peak and average 2D axial WSS and circumferential WSS were measured in the MPA at the same plane as that used for the calculation of the 3D WSS. Regional variations were also assessed by creating 8 equal segments around the circumference of the MPA and measuring WSS_ax_ and WSS_circ_ in each segment Figure 2.

**Figure 2:**
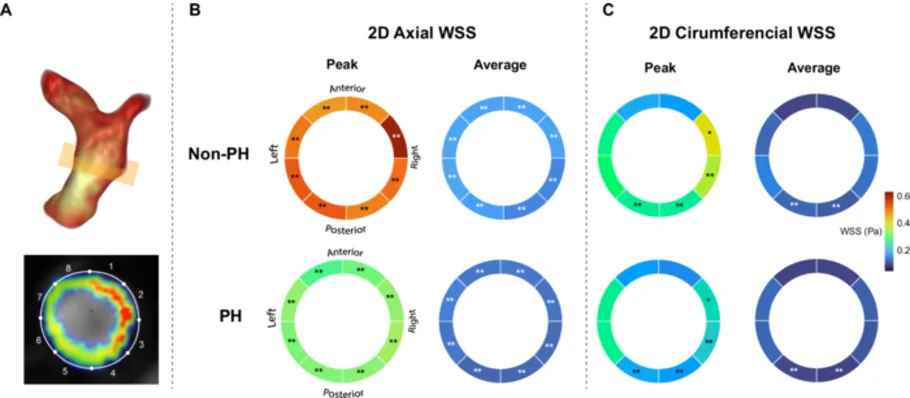
Main pulmonary artery regional component WSS. Depiction of differences in axial (B) and circumferential (C) components of peak and average WSS in non‐PH (upper rings) and PH patients (lower rings). Individual segments depict equal arcs around the wall of the MPA at the level 1 cm above the pulmonary valve (A). Asterisks denote statistically significant differences in respective segments between PH and non‐PH cohorts. *p < 0.05, **p < 0.005, MPA = main pulmonary artery, Pa = Pascals, PH = pulmonary hypertension, WSS = wall shear stress.

Volumetric analysis of the CMR cines was performed by an operator (DG) with 19 years of CMR experience. The 4D‐flow and CMR strain analysis was performed by an operator (BS) with 11 years of CMR experience. To assess inter‐operator reproducibility a subset of 20 of 4D‐flow and strain datasets were independently analyzed by 2nd operator (KL) blinded to the original results. All analysis of 4D‐flow and CMR images was performed blind to the hemodynamic results of the RHC. Images from subjects in both the validation and test cohort underwent this same MRI analysis.

### 2.4 Computation Fluid Dynamics Analysis

To address the tendency of 4D‐flow MRI to underestimate WSS due to low spatial resolution [32], we validated a subset of patient data using computational fluid dynamics (CFD). We randomly selected 16 patients for patient‐specific CFD analysis via Ansys CFX (v25). Following the methodology presented in our previous study [26], each CFD model was built based on the patient's 4D‐flow data. In summary, 3D geometries were segmented using all three velocity phase images as well as the magnitude images to ensure the complete PA was captured, and geometries were meshed in Ansys ICEM. In‐house Python codes were used to process the 4D‐flow MRI data and evaluate velocity data, allowing for a 3D time‐varying inlet velocity profile at the level of the pulmonary valve to be extracted for each case [33]. Additionally, flow distribution between the left and right pulmonary arteries was quantified by calculating the proportion of main pulmonary artery inflow entering each branch over the cardiac cycle. These time‐integrated flow fractions were then prescribed as outlet boundary conditions, such that each outlet received the corresponding percentage of the inlet flow. Rather than applying branch‐specific waveforms directly to the outlets, the total flow split was used as noise from the scan can introduce inconsistencies with mass conservation and lead to non‐physical flow imbalances within the CFD simulation.

Demonstration of a strong quantitative and qualitative agreement in velocity fields between our patient‐specific CFD models and corresponding 4D‐flow MRI data allowed us to confidently interpret velocity derivative parameters such as WSS_avg_ directly from the CFD [26]. To take advantage of the high spatial resolution, the CFD predicted WSS_avg_ was evaluated as an average across the entire MPA surface. As our previous work and the 4D‐flow results of this study highlighted the important influence of the flow splitting into the LPA and RPA, this surface of analysis included the bifurcation and the first 1 cm of both branches.

### 2.5 Statistical Analysis

Continuous data with a normal distribution are presented as mean (±1 standard deviation (SD)), and non‐normally distributed data are presented as median (inter‐quartile range). Comparison of continuous data in the validation cohort between the PH and healthy controls was performed with an independent sample two‐tailed Student's *t* test or Mann–Whitney U as appropriate. Where statistically significant differences existed between the demographics of the two groups, linear regression was performed to control for these differences.

In the validation cohort, correlation analysis was performed using Pearson's correlation coefficient or Spearman's rank test, where the data were non‐normal. Receiver operating characteristic (ROC) curves with area under the curve (AUC) values were used to assess the discriminative ability of WSS to predict PH and RV‐PA uncoupling in the validation cohort. The optimal cut‐points were defined by the Youden index. Intraclass correlation coefficient (ICC) estimates and their 95% confidence intervals for interobserver variability in 4D‐flow WSS and CMR strain measurements were also calculated. For all statistical tests performed, a *p*‐value of ≤ 0.05 was considered statistically significant.

In the test cohort, the sensitivity, specificity, positive predictive value (PPV), and negative predictive value (NPV) were calculated for the identification of PH and RV‐PA uncoupling using the optimum cutoff values for WSS calculated from the validation cohort. Statistical analysis was conducted using R version 4.4.2 (R Core Team, 2024) in RStudio Version 2024.09.1 + 394 (Rstudio Team, 2024).

## 3 Results

In the validation cohort, 46 patients and 20 health controls were recruited; 2 subjects were later excluded from the study due to technical reasons (1 poor image quality due to arrhythmia during MRI acquisition and 1 where the 4D‐flow raw data were corrupted). At RHC, 3 patients recorded an mPAP of < 20 and were subsequently included in the non‐PH group along with the healthy controls. Consequently, 41 subjects with PH and 23 subjects without PH were included in the validation study. Figure 1 summarizes the patient flow and study design, and Table 1 shows subject demographics, CMR metric, and patient hemodynamic results from both validation and test cohorts.

**Table 1:** Demographics, hemodynamic parameters from RHC and functional parameters from cardiac MRI for PH and non‐PH participants in both the validation and test cohorts.

Patient and volunteer demographics						
	Validation cohort			Test cohort		
	non‐PH N = 23^a^	PH = N = 41^a^	*p*‐value	non‐PH N = 18^a^	PH N = 22^a^	*p*‐value
Age	42 (13)	56 (14)	***	35 (12)	50 (17)	*
Gender			NS			NS
F	13 (57%)	29 (71%)		9 (50%)	10 (45%)	
M	10 (43%)	12 (29%)		9 (50%)	12 (55%)	
BMI	26.7 (3.9)	27.2 (6.5)	NS	23.6 (3.3)	27.6 (4.7)	**
PH subtype						
HVOL	20			15		
No PH	3			3		
PAH		24			15	
LHD		1			1	
Lung disease		1			1	
CTEPH		15			5	
Hemodynamic parameters						
RHC delay		5 (7)			2 (1)	
mPAP (mmHg)	18 (1)^c^	44 (12)	**	17 (3)^c^	41 (12)	**
mPCWP (mmHg)	8 (4)^c^	10 (4)	NS	8 (4)^c^	10 (4)	NS
PVR (WU)	2.1 (0.7)^c^	9.5 (5.9)	**	1.5 (0.8)^c^	7.2 (4.0)	**
CO (l/m)	4.5 (0.51)^c^	4.1 (1.26)	NS	6.9 (3.57)^c^	5.0 (2.05)	NS
CMR parameters						
LVEF	65 (4)	64 (9)	NS	67 (6)	63 (7)	NS
LV GLS (%)	−18.2 (2.5)	‐16.4 (3.3)	*	−17.76 (2.6)	−16.22 (3.3)	NS
LA GLS (%)	46 (16)	26 (13)	***	39 (15)	28 (13)	**
RVEDVi	77 (14)	112 (36)	***	89 (21)	100 (28)	NS
RVESVi	30 (8)	65 (30)	***	37 (9)	57 (21)	**
RVSVi	47 (8)	47 (17)	NS	53 (13)	44 (14)	*
RVEF	61 (5)	44 (12)	***	59 (3)	45 (11)	***
RV GLS (%)	−24.4 (4)	‐17.4 (5)	***	−23.9 (4)	−15.9 (5)	***
RA GLS (%)	52 (24)	27 (13)	***	44 (24)	24 (11)	**

^a^ Mean (SD)

^b^ Mann‐Whitney *U* test.

^c^ Hemodynamic values only measured in patients undergoing RHC who were referred with suspected PH but later reclassified as non‐PH. Healthy volunteers did not undergo RHC.

Significance levels: *p < 0.05, **p < 0.01, ***p < 0.001.

Abbreviations: BMI = body mass index, CO = cardiac output, CTEPH = chronic thromboembolic pulmonary hypertension, GLS = global longitudinal strain, HVOL = healthy volunteer, l/m = liters per minute, LA = left atrium, LHD = left heart disease, LV = left ventricle, LVEF = left ventricle ejection fraction, mmHg = millimeters of mercury, mPAP = mean pulmonary artery pressure, mPCWP = mean pulmonary capillary wedge pressure, MRI = magnetic resonance imaging, PAH = pulmonary arterial hypertension, PH = pulmonary hypertension, PVR = pulmonary vascular resistance, RA = right atrium, RHC = right heart catheterization, RV = right ventricle, RVEDVi = indexed right ventricle end diastolic volume, RVEF = right ventricle ejection fraction, RVESVi = indexed right ventricle end systolic volume, RVSVi = indexed right ventricle stroke volume, SD = standard deviation, WU = Wood's units.

The mean PH patient age was 56 ± 14 years with a female preponderance (71%). The average delay between the MRI and RHC for this validation study was 5 (±7) days. The PH patient cohort had significant elevation of mPAP (44 ± 12 mmHg) and pulmonary vascular resistance (PVR 9.5 ± 5.9 WU) with preserved left ventricular (LV) but impaired right ventricular (RV) systolic function. Pulmonary arterial hypertension (PAH, 24/41, 59%) and chronic thromboembolic PH (CTEPH, 37%) were the most common PH diagnoses. All healthy volunteers (mean age 42 ± 13 years, 57% female) had normal biventricular size and systolic function.

### 3.1 Wall Shear Stress

Both 3D WSS_peak_ and 3D WSS_avg_ in the MPA, LPA, and RPA were significantly reduced in PH patients compared to healthy controls: these differences retained significance when controlling for age and gender. The global measures of 3D WSS_global_peak_ and 3D WSS_global_avg_ were 0.43 ± 0.1 and 0.15 ± 0.03, respectively, in the healthy volunteers and 0.17 ± 0.06 and 0.07 ± 0.02, respectively, in the PH cohort (Table 2; Supporting Information S1: Figure S1).

**Table 2:** Comparison of 3D and 2D wall shear stress measurements in the PH and non‐PH cohorts in the validation cohort.

Wall shear stress measures from 4D‐flow			
	**non‐PH** N = **23** ^**1**^	**PH N** = **41** ^**1**^	***p*‐value** ^**2**^
Main pulmonary artery			
3D WSS_peak_	0.37 (0.09)	0.20 (0.07)	< 0.001
3D WSS_avg_	0.12 (0.03)	0.08 (0.02)	< 0.001
Axial 2D WSS_peak_	0.44 (0.13)	0.26 (0.10)	< 0.001
Axial 2D WSS_avg_	0.14 (0.03)	0.10 (0.03)	< 0.001
Circumferential 2D WSS_peak_	0.19 (0.06)	0.13 (0.06)	< 0.001
Circumferential 2D WSS_avg_	0.09 (0.03)	0.07 (0.03)	0.001
Right pulmonary artery			
3D WSS_peak_	0.47 (0.15)	0.17 (0.07)	< 0.001
3D WSS_avg_	0.16 (0.04)	0.08 (0.03)	< 0.001
Left pulmonary artery			
3D WSS_peak_	0.44 (0.13)	0.15 (0.06)	< 0.001
3D WSS_avg_	0.15 (0.04)	0.07 (0.03)	< 0.001
Global wall shear stress			
3D WSS_global_peak_	0.43 (0.10)	0.17 (0.06)	< 0.001
3D WSS_global_avg_	0.15 (0.03)	0.07 (0.02)	< 0.001

*Note:* All WSS measurements in Pascals (Pa). ^1^Mean (SD), ^2^Mann–Whitney *U* test.

Abbreviations: 3D WSS_global_avg_ = average wall shear stress (spatially average across all three vessels), 3D WSS_global_peak_ = peak wall shear stress (spatially average across all three vessels), PH = pulmonary hypertension, WSS_avg_ = average wall shear stress (single vessel), WSS_peak_ = peak wall shear stress (single vessel).

Both 2D axial WSS_peak_ and WSS_avg_ and circumferential 2D WSS_peak_ and WSS_avg_ were significantly reduced in the MPA of patients with PH compared with the healthy controls. (Table 2). The regional wall analysis of axial WSS_peak_ and WSS_avg_ in the MPA showed significantly reduced values in all segments for PH patients, while the circumferential WSS_peak_ and WSS_avg_ was significantly reduced for PH patients along the right (segments 2 and 3) and posterior (segments 4 and 5) walls (Figure 2). The relative contribution of the circumferential component to overall 3D WSS magnitude was greater in PH patients than in the non‑PH cohort, more significantly along the lateral walls of the MPA.

There was no statistically significant difference in any of the 2D or 3D WSS measures between the patients with CTEPH and those with PAH (Supporting Information S1: Figure S2).

### 3.2 Correlation Between WSS and Hemodynamic Parameters

Table 3 and Figure 3 show the correlations between the WSS measures and the hemodynamic measures from the RHC examination. The strongest correlations with the mPAP were for the global measure of 3D WSS_global_peak_ (*r* = −0.75, *p* < 0.001) and 3D WSS_peak_ in the RPA (*r* = −0.70, *p* < 0.001) and LPA (r = −0.69, *p* < 0.001). The strongest correlations with the PVR was the 3D WSS_global_peak_ (*r* = −0.76, *p* < 0.001) and 3D WSS_global_avg_ (*r* = −0.73, *p* < 0.001). None of the WSS measures showed any correlation with the mPCWP.

**Table 3:** Pearson correlation (*r*) between 4D flow measured wall shear stress and RHC measured hemodynamics in the validation cohort.

Correlation of 4D‐flow WSS and invasive hemodynamics			
	**mPAP**	**PVR**	**mPCWP**
Main pulmonary artery			
3D WSS_peak_	−0.61***	−0.61***	0.25
3D WSS_avg_	−0.47***	−0.54***	0.18
Axial 2D WSS_peak_	−0.49***	−0.55***	0.19
Axial 2D WSS_avg_	−0.38*	−0.48**	0.12
Circumferential 2D WSS_peak_	−0.39*	−0.48**	0.16
Circumferential 2D WSS_avg_	−0.16	−0.33	0.08
Right pulmonary artery			
3D WSS_peak_	−0.70***	−0.70***	−0.03
3D WSS_avg_	−0.56***	−0.62***	−0.02
Left pulmonary artery			
3D WSS_peak_	−0.69***	−0.71***	0.04
3D WSS_avg_	−0.68***	−0.73***	0.05
Global wall shear stress			
3D WSS_global_peak_	−0.75***	−0.76***	0.04
3D WSS_global_avg_	−0.69***	−0.73***	0.07

Significance levels: *p < 0.05,**p < 0.01, ***p < 0.001.

Abbreviations: 3D WSS_global_avg_ = average wall shear stress (spatially average across all three vessels), 3D WSS_global_peak_ = peak wall shear stress (spatially average across all three vessels), mPAP = mean pulmonary artery pressure, mPCWP = mean pulmonary capillary wedge pressure, PH = pulmonary hypertension, PVR = pulmonary vascular resistance, WSS_avg_ = average wall shear stress (single vessel), WSS_peak_ = peak wall shear stress (single vessel).

**Figure 3:**
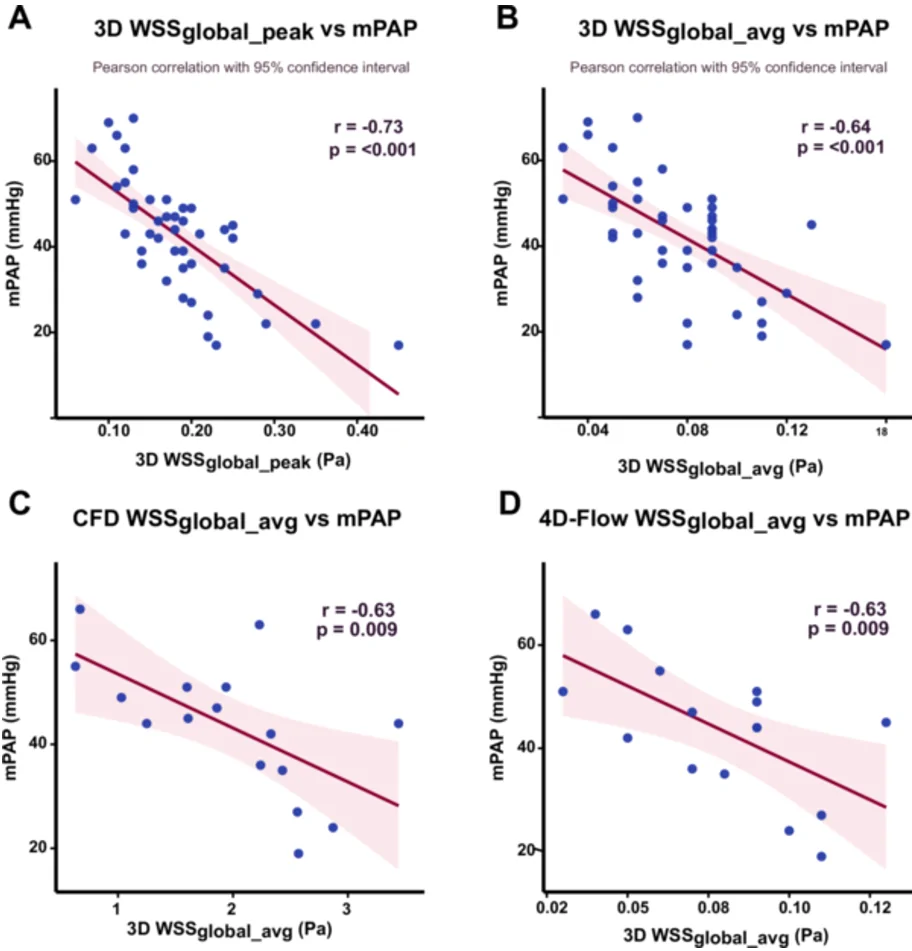
Correlation Plots. Upper row: Pearson's correlation plot between 3D WSS_global_peak_ and mPAP (A) and 3D WSS_global_avg_ (B) and mPAP from all PH patients in the test cohort. Lower row: the subset of 16 PH patients who underwent CFD showing Pearson's correlation plots for CFD‐derived WSS_global_avg_ and mPAP (C), and 4D flow‐derived WSS_global_avg_ and mPAP (D). CFD = computational fluid dynamics, mPAP = mean pulmonary artery pressure, Pa = Pascals, WSS_global_avg_ = average wall shear stress, WSS_global_peak_ = peak wall shear stress.

Results from the CFD subset of patients showed a strong correlation between mPAP and WSS_avg_ averaged over the single surface that covered the MPA, RPA, and LPA (*r* = −0.63, *p* < 0.01), a result that mirrored the correlation between mPAP and the 4D‐Flow derived 3D WSS_global_avg_ in the same subset of 16 patients (*r* = −0.63, *p* < 0.01) (Figure 3). This evaluation of the CFD‐derived WSS_avg_ values validates and confirms confidence in the findings of the results directly from the 4D‐flow data.

### 3.3 Correlation Between WSS and CMR Functional Parameters

Correlation with the RVEF was strongest for 3D WSS_peak_ in the MPA (*r* = −0.69, *p* < 0.001) and the global measure of 3D WSS_global_peak_ (*r* = −0.62, *p* < 0.001). For the RV GLS, 3D WSS_global_avg_ showed a good correlation (*r* = −0.61, *p* < 0.001). Supporting Information S1: Table S1 shows the correlations between all 4D‐flow WSS measures and the functional CMR parameters for both PH patients and healthy volunteers.

### 3.4 Prediction of PH

Using the updated hemodynamic thresholds for PH prediction (mPAP > 20 mmHg and PVR > 2WU), ROC curve analysis demonstrated that WSS was able to accurately distinguish PH patients from healthy controls (Figure 4; Supporting Information S1: Table S1). Among all parameters, 3D WSS_peak_ in the RPA (cutoff < 0.29 Pa, AUC 0.99) and 3D WSS_global_peak_ (cutoff < 0.3 Pa, AUC 0.98) performed best at predicting patients with an mPAP> 20 mmHg and PVR > 2WU.

**Figure 4:**
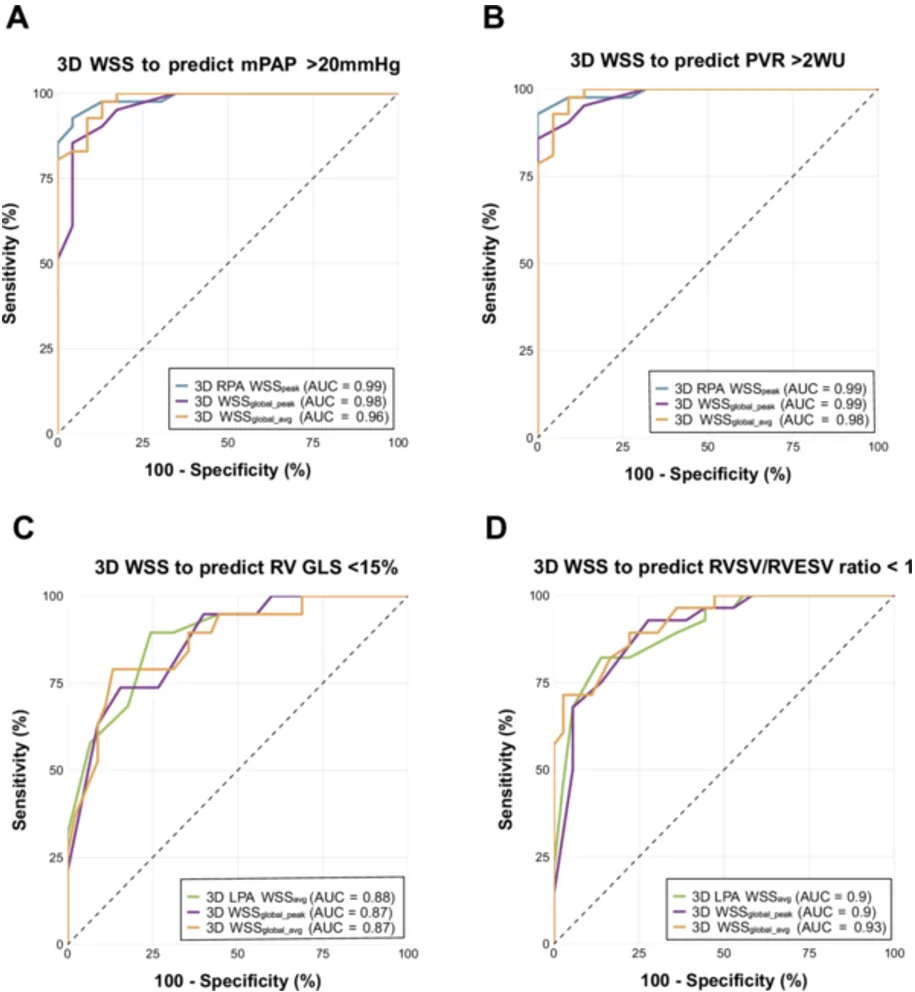
Receiver operating characteristic curves. Receiver operating characteristic curves demonstrate the ability of WSS measures in the validation cohort to predict an mPAP > 20 mmHg (A), PVR > 2WU (B), RV GLS < 15.3% (C), and an RVSV/RVESV ratio < 1 (D). Supporting Information S1: Table S2 gives further details of 95% CI, sensitivity, and specificity. AUC = area under the curve, CI = confidence interval, GLS = global longitudinal strain, LPA = left pulmonary artery, mmHg = millimeters of mercury, mPAP = mean pulmonary artery pressure, PVR = pulmonary vascular resistance, RPA = right pulmonary artery, RV = right ventricle, RVESV = right ventricular end systolic volume, RVSV = right ventricular stroke volume, WSS = wall shear stress.

### 3.5 Prediction of Right Ventricle–Pulmonary Artery Uncoupling

To identify subjects with RV‐PA uncoupling, the previously published thresholds of RV global longitudinal strain (GLS) < −15.3% and volumetric approximations (RVSV/RVESV < 1) were used [18, 19]. ROC curve analysis (Figure 4, Supporting Table S1) identified a WSS_global_peak_ value of 0.18 Pa as an excellent predictor of both a GLS < −15.3 (AUC 0.87) and RVSV/SVESV < 1 (AUC 0.93).

### 3.6 Repeatability Assessment

The ICC for 3D WSS and RV strain functional parameters were 0.98 (*p* < 0.05) and 0.71 (*p* < 0.05), respectively.

### 3.7 Test Cohort

In the test cohort, 24 patients and 17 healthy controls were recruited: 1 patient was later excluded due to poor 4D‐flow image quality due to arrhythmia. The average delay between the MRI and RHC for the test study was 2 (±1) days.

Using a cutoff value of < 0.3 Pa from the validation study, the 3D WSS_global_peak_ was correctly able to identify patients with PH with a sensitivity of 90% and a specificity of 100%. There were two false negative results, both of whom had a WSS_global_peak_ of 0.32 Pa; one patient had an mPAP = 26 mmHg and PVR = 4.7WU and another had mPAP = 27 mmHg and PVR = 3.2WU at RHC (Figure 5).

**Figure 5:**
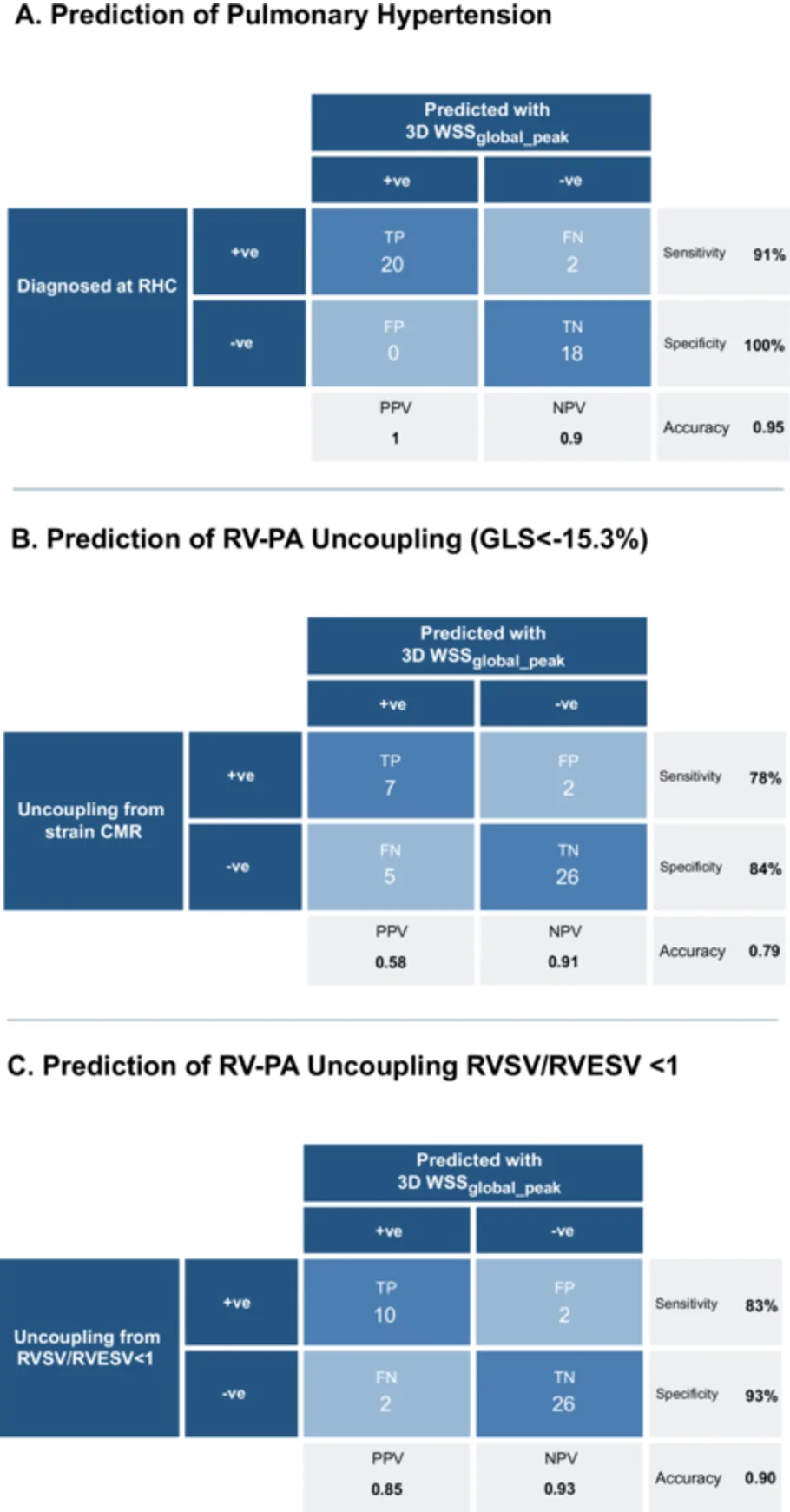
Confusion matrices. Confusion matrices for 3D WSS_global_peak_ to identify an mPAP > 20 (A) and RV‐PA uncoupling using the CMR‐derived metric of GLS < −15.3%(B) and RV‐PA uncoupling using the CMR‐derived metric of RVSV/RVESV < 1 (C) in the test cohort. CMR = cardiovascular magnetic resonance, FN = false negative, FP = false positive, GLS = global longitudinal strain, NPV = negative predictive value, PPV = positive predictive value, RHC = right heart catheterization, RV‐PA = right ventricle pulmonary artery, RVESV = right ventricular end systolic volume, RVSV = right ventricular stroke volume, TN = true negative, TP = true positive, WSS_global_peak_ = peak wall shear stress.

A 3D WSS_global_peak_ cutoff value of < 0.18 Pa was able to correctly identify RV‐PA uncoupling as measured by RV strain (GLS < −15.3%) with a sensitivity of 78% and specificity of 84%, and uncoupling as measured by an RVSV/SVESV ratio < 1 with a sensitivity of 83% and a specificity of 93% (Figure 5).

## 4 Discussion

This study demonstrates 4D‐flow MRI‐derived WSS to be a strong non‐invasive biomarker for both the detection of PH and the prediction of RV‐PA uncoupling. To our knowledge, this represents the largest 4D‐flow MRI investigation of pulmonary arterial WSS to date, and the first to apply the updated 2022 ESC/ERS hemodynamic definition of PH, incorporating both a mPAP threshold of > 20 mmHg and a PVR ≥ 2 Wood units as the diagnostic reference standard.

Consistent with previous studies, we observed significant reductions in both peak systolic and time‐averaged WSS across the main, right, and left pulmonary arteries in PH patients compared to healthy controls, with differences persisting after controlling for age and gender [9, 11, 12]. The global composite metrics, WSS_global_peak_ and WSS_global_avg_, demonstrated the strongest correlations with invasive hemodynamic parameters, with WSS_global_peak_ correlating strongly with both mPAP and PVR. These results were supported by the high‐fidelity CFD and reinforce those of Armour et al., by demonstrating that a comparable spatial averaging approach applied directly to 4D‐flow MRI data yields similarly strong associations across a substantially larger and more clinically diverse cohort [26].

A novel contribution of our study is the characterization of the directional components of WSS in the proximal pulmonary arteries. Axial WSS was uniformly and significantly reduced across all eight segments of the MPA in PH patients. In contrast, circumferential WSS reduction was heterogeneous, the most pronounced reductions along the right and posterior walls of the MPA. Despite these absolute reductions in circumferential WSS, its relative contribution to overall WSS magnitude was proportionally greater in PH patients than in controls. This change in the directional components of shear forces likely reflects the emergence of complex, vortical flow patterns seen in the presence of raised mPAP and vessel dilatation [34], and may have implications for the endothelial dysfunction and vascular remodeling observed in PH.

The diagnostic performance of WSS for PH detection was strong. The optimum WSS_global_peak_ cutoff identified in the validation cohort yielded a sensitivity of 90% and specificity of 100%, with an overall accuracy of 0.95 when applied in an independent test cohort. The two false negative cases both had mildly elevated pressures (mPAP 26–27 mmHg) and recorded WSS_global_peak_ values of 0.32 Pa, marginally above the threshold. Refinement of the WSS cutoff value or incorporation of additional hemodynamic parameters may be required to improve sensitivity at the diagnostic boundary.

Beyond PH detection, this study establishes WSS as a potential predictor of RV‐PA uncoupling, a critical prognostic transition in PH pathophysiology. A WSS_global_peak_ threshold of < 0.18 Pa demonstrated excellent discriminative ability for both CMR‐derived markers of uncoupling: RV global longitudinal strain < −15.3% (AUC 0.87) and RVSV/RVESV ratio < 1 (AUC 0.93). In the test cohort, this threshold identified volumetric uncoupling with a sensitivity of 83% and specificity of 93%. The strong correlations between WSS and RV ejection fraction and RV GLS suggest a mechanistic link between impaired pulmonary arterial hemodynamics and progressive RV dysfunction. These data suggest that a single integrated MRI examination incorporating 4D‐flow WSS analysis and cine‐derived RV functional metrics could provide a comprehensive, non‐invasive assessment of both disease presence and severity of RV‐PA coupling.

The excellent inter‐observer reproducibility of 3D WSS measurements (ICC = 0.98) supports the technical robustness of the spatial averaging methodology and addresses a key methodological concern in the field regarding the sensitivity of WSS estimates to operator‐dependent plane placement [35]. This high reproducibility, combined with the strong diagnostic performance validated in a prospective test cohort, strengthens the translational potential of WSS as a clinically useful biomarker.

There were several limitations within our study. Firstly, the high AUC values we reported for PH prediction are presented with the caveat of spectrum bias. The study design primarily compared healthy volunteers against a cohort with established, hemodynamically significant disease (mean mPAP = 44 mmHg), rather than a population of undifferentiated patients with suspected PH. Consequently, these results will overstate the diagnostic performance of WSS in a real‐world clinical setting, where the primary challenge lies in differentiating borderline or early‐stage PH from symptomatic non‐PH patients

The use of a global WSS measure assumed equal weighting across the three vessels analyzed, which could mask hemodynamic asymmetries possible in CTEPH. Nevertheless, our sub‐analysis demonstrated no significant difference in RPA and LPA WSS values between the PAH and CTEPH subgroups, although these findings must be interpreted with caution, given the small numbers in each of these subgroups.

The study was conducted at a single center using a 1.5 T scanner with a prototype 4D‐flow sequence, and the generalizability of the derived cutoff values to other field strengths, scanner platforms, and post‐processing software requires prospective evaluation. As acknowledged in the literature, 4D‐flow MRI systematically underestimates WSS magnitudes relative to CFD‐derived values [24, 25]. The diagnostic WSS cutoff established in this study must be interpreted within the context of resolution‐dependent measurement uncertainty. The impact of spatial resolution and segmentation accuracy are inherent source of imprecision in 4D‐flow WSS estimation, where typical voxel sizes used in clinical practice may underestimate WSS by up to 70% due to the partial volume smoothing of the near‐wall velocity gradient [36]. However, despite this underestimation, regions of reduced WSS have been shown to agree across different resolutions in both CFD and in vivo studies [24, 37] and our results demonstrated that the correlation between 3D WSS and mPAP was consistent between the 4D‐flow and high‐resolution CFD. Nevertheless, it is important to bear in mind that the absolute thresholds derived in our study remain relative to the spatial resolution of the 4D‐sequence used and the post‐processing segmentation performed.

The heterogeneity of PH subtypes within the cohort, while reflective of real‐world clinical practice, may introduce variability in WSS profiles that warrants subgroup analysis in future studies. Finally, the surrogate CMR markers of RV‐PA coupling used in this study, while validated, represent indirect approximations of the reference‐standard invasive pressure‐volume loop methodology.

In summary, 4D‐flow MRI‐derived spatially averaged global peak WSS offers promising diagnostic accuracy for detecting PH under the updated hemodynamic definition of the disease and a meaningful predictive marker of RV‐PA uncoupling. These findings support the integration of WSS analysis into routine CMR protocols for PH, offering a non‐invasive complement to right heart catheterization that may facilitate earlier diagnosis and a biomarker to monitor during follow‐up examinations.

## Author Contributions

**Ben Statton:** conceptualization, methodology, data acquisition, data pre‐processing, data analysis, writing – original draft, writing – review and editing. **Chlöe Armour:** methodology, data pre‐processing, data analysis, writing – original draft, writing – review and editing. **Kai'en Leong:** conceptualization, methodology, data analysis, writing – original draft, writing – review and editing. **Jan Sedlacik:** software, data curation, data pre‐processing, data analysis, writing – review and editing. **Alaine Berry:** data acquisition, data curation, writing – review and editing. **Ning Jin:** software, writing – review and editing. **Daniel Giese:** software, writing – review and editing. **Francesco Lo Guidice:** data acquisition, writing – review and editing. **Luke Howard:** data acquisition, writing – review and editing. **Deepa Gopalan:** supervision, conceptualization, methodology, data analysis, writing – original draft, writing – review and editing.

## Ethics Statement

The study was performed in accordance with the Health Research Authority, Health and Care Research Wales (HCRW) Approval (Reference 20/HH/5838); Health Research Authority, South Central – Hampshire B Research Ethics Committee (Reference 21/SC/0239); and Health Research Authority – East of England –Cambridgeshire and Hertfordshire Research Ethics Committee (Reference 17/EE/0068).

## Conflicts of Interest

The authors declare no conflicts of interest.

## Supporting information


Supporting File

## Data Availability

The data that support the findings of this study are available from the corresponding author upon reasonable request.

## References

[1] G. Kovacs , S. Bartolome , C. P. Denton , et al., “Definition, Classification and Diagnosis of Pulmonary Hypertension,” European Respiratory Journal 64 (2024): 2401324, 10.1183/13993003.01324-2024. PMC11533989 39209475

[2] M. Humbert , G. Kovacs , M. M. Hoeper , et al., “2022 ESC/ERS Guidelines for the Diagnosis and Treatment of Pulmonary Hypertension,” European Heart Journal 43 (2022): 3618–3731, 10.1093/eurheartj/ehac237. 36017548

[3] G. Simonneau , D. Montani , D. S. Celermajer , et al., “Haemodynamic Definitions and Updated Clinical Classification of Pulmonary Hypertension,” European Respiratory Journal 53 (2019): 1801913, 10.1183/13993003.01913-2018. PMC6351336 30545968

[4] G. M. B. Meyer , F. B. Spilimbergo , S. Atmayer , et al., “Multiparametric Magnetic Resonance Imaging in the Assessment of Pulmonary Hypertension: Initial Experience of a One‐Stop Study,” Lung 196 (2018): 165–171, 10.1007/s00408-018-0097-7. 29435739

[5] L. C. Saunders , P. J. C. Hughes , S. Alabed , et al., “Integrated Cardiopulmonary MRI Assessment of Pulmonary Hypertension,” Journal of Magnetic Resonance Imaging 55 (2022): 633–652, 10.1002/jmri.27849. 34350655

[6] G. Reiter , C. Reiter , I. Ovcina , M. Fuchsjäger , and U. Reiter , “Four‐Dimensional Flow MRI for a Dynamic Perspective on the Heart and Adjacent Great Vessels,” Radiology 316 (2025): e242972, 10.1148/radiol.242972. 40793945

[7] G. Reiter , U. Reiter , G. Kovacs , et al., “Magnetic Resonance–Derived 3‐Dimensional Blood Flow Patterns in the Main Pulmonary Artery as a Marker of Pulmonary Hypertension and a Measure of Elevated Mean Pulmonary Arterial Pressure,” Circulation. Cardiovascular imaging 1 (2008): 23–30, 10.1161/circimaging.108.780247. 19808511

[8] J. R. Kroeger , M. Stackl , K. Weiss , et al., “K‐T Accelerated Multi‐VENC 4D Flow MRI Improves Vortex Assessment in Pulmonary Hypertension,” European Journal of Radiology 145 (2021): 110035, 10.1016/j.ejrad.2021.110035. 34801875

[9] A. J. Barker , A. Roldán‐Alzate , P. Entezari , et al., “Four‐Dimensional Flow Assessment of Pulmonary Artery Flow and Wall Shear Stress in Adult Pulmonary Arterial Hypertension: Results From Two Institutions,” Magnetic Resonance in Medicine 73 (2015): 1904–1913, 10.1002/mrm.25326. PMC4277737 24974951

[10] K. Odagiri , N. Inui , A. Hakamata , et al., “Non‐Invasive Evaluation of Pulmonary Arterial Blood Flow and Wall Shear Stress in Pulmonary Arterial Hypertension With 3D Phase Contrast Magnetic Resonance Imaging,” SpringerPlus 5 (2016): 1071, 10.1186/s40064-016-2755-7. PMC4943915 27462519

[11] M. Schäfer , V. O. Kheyfets , J. D. Schroeder , et al., “Main Pulmonary Arterial Wall Shear Stress Correlates With Invasive Hemodynamics and Stiffness in Pulmonary Hypertension,” Pulmonary Circulation 6 (2016): 37–45, 10.1086/685024. PMC4809665 27076906

[12] M. Terada , Y. Takehara , H. Isoda , et al., “Low Wss and High Osi Measured by 3D Cine Pc Mri Reflect High Pulmonary Artery Pressures in Suspected Secondary Pulmonary Arterial Hypertension,” Magn Reson Méd Sci 2015 15: 193–202, 10.2463/mrms.mp.2015-0038. PMC5600056 26567758

[13] P. F. Davies , “Hemodynamic Shear Stress and the Endothelium in Cardiovascular Pathophysiology,” Nature Clinical Practice Cardiovascular Medicine 6 (2009): 16–26, 10.1038/ncpcardio1397. PMC2851404 19029993

[14] E. Tzima , M. Irani‐Tehrani , W. B. Kiosses , et al., “A Mechanosensory Complex That Mediates the Endothelial Cell Response to Fluid Shear Stress,” Nature 437 (2005): 426–431, 10.1038/nature03952. 16163360

[15] B. T. Tang , S. S. Pickard , F. P. Chan , P. S. Tsao , C. A. Taylor , and J. A. Feinstein , “Wall Shear Stress Is Decreased in the Pulmonary Arteries of Patients With Pulmonary Arterial Hypertension: An Image‐Based, Computational Fluid Dynamics Study,” Pulmonary Circulation 2 (2012): 470–476, 10.4103/2045-8932.105035. PMC3555417 23372931

[16] A. Vonk Noordegraaf , B. E. Westerhof , and N. Westerhof , “The Relationship Between the Right Ventricle and Its Load in Pulmonary Hypertension,” Journal of the American College of Cardiology 69 (2017): 236–243, 10.1016/j.jacc.2016.10.047. 28081831

[17] P. Trip , S. Rain , M. L. Handoko , et al., “Clinical Relevance of Right Ventricular Diastolic Stiffness in Pulmonary Hypertension,” European Respiratory Journal 45 (2015): 1603–1612, 10.1183/09031936.00156714. 25882798

[18] K. Tello , A. Dalmer , R. Vanderpool , et al., “Cardiac Magnetic Resonance Imaging‐Based Right Ventricular Strain Analysis for Assessment of Coupling and Diastolic Function in Pulmonary Hypertension,” JACC. Cardiovascular Imaging 12 (2019): 2155–2164, 10.1016/j.jcmg.2018.12.032. 30878422

[19] J. Sanz , A. García‐Alvarez , L. Fernández‐Friera , et al., “Right Ventriculo‐Arterial Coupling in Pulmonary Hypertension: A Magnetic Resonance Study,” Heart 98 (2012): 238–243, 10.1136/heartjnl-2011-300462. 21917658

[20] M. Schäfer , D. D. Ivy , S. H. Abman , et al., “Differences in Pulmonary Arterial Flow Hemodynamics Between Children and Adults With Pulmonary Arterial Hypertension as Assessed by 4D‐flow CMR Studies,” American Journal of Physiology‐Heart and Circulatory Physiology 316 (2019): H1091–H1104, 10.1152/ajpheart.00802.2018. PMC7327229 30822118

[21] H. Koike , T. Nishimura , and M. Morikawa , “Quantitative Evaluation of Pulmonary Hypertension Using 4D Flow MRI: A Retrospective Study,” Heliyon 10 (2024): e31177, 10.1016/j.heliyon.2024.e31177. PMC11133668 38813238

[22] B. J. Kim , J. Lee , H. Berhane , B. H. Freed , S. J. Shah , and J. D. Thomas , “Differences in Pulmonary Artery Flow Hemodynamics Between PAH and PH‐HFpEF: Insights From 4D‐Flow CMR,” Pulmonary Circulation 15 (2025): e70022, 10.1002/pul2.70022. PMC11693845 39749111

[23] K. Ohno , K. Suwa , K. Akita , R. Sato , T. Satoh , and Y. Maekawa , “Hemodynamics Assessment Using 4D Flow CMR before and After Balloon Pulmonary Angioplasty in Chronic Thromboembolic Pulmonary Hypertension: A Retrospective Observational Study,” BMC Cardiovascular Disorders 25 (2025): 621, 10.1186/s12872-025-05068-x. PMC12369117 40841600

[24] M. Cibis , W. V. Potters , F. J. Gijsen , et al., “The Effect of Spatial and Temporal Resolution of Cine Phase Contrast MRI on Wall Shear Stress and Oscillatory Shear Index Assessment,” PLoS One 11 (2016): e0163316, 10.1371/journal.pone.0163316. PMC5036833 27669568

[25] J. Zhang , S. M. Rothenberger , M. C. Brindise , M. Markl , V. L. Rayz , and P. P. Vlachos , “Wall Shear Stress Estimation for 4D Flow MRI Using Navier–Stokes Equation Correction,” Annals of Biomedical Engineering 50 (2022): 1810–1825, 10.1007/s10439-022-02993-2. PMC10263099 35943617

[26] C. H. Armour , D. Gopalan , B. Statton , et al., “Patient‐Specific Modelling of Pulmonary Arterial Hypertension: Wall Shear Stress Correlates With Disease Severity,” Frontiers in Bioengineering and Biotechnology 13 (2025): 1585345, 10.3389/fbioe.2025.1585345. PMC12213805 40606912

[27] M. A. Griswold , P. M. Jakob , R. M. Heidemann , et al., “Generalized Autocalibrating Partially Parallel Acquisitions (GRAPPA),” Magnetic Resonance in Medicine 47 (2002): 1202–1210, 10.1002/mrm.10171. 12111967

[28] M. Markl , A. Harloff , T. A. Bley , et al., “Time‐Resolved 3D MR Velocity Mapping at 3T: Improved Navigator‐Gated Assessment of Vascular Anatomy and Blood Flow,” Journal of Magnetic Resonance Imaging 25 (2007): 824–831, 10.1002/jmri.20871. 17345635

[29] M. M. Bissell , F. Raimondi , L. Ait Ali , et al., “4D Flow Cardiovascular Magnetic Resonance Consensus Statement: 2023 Update,” Journal of Cardiovascular Magnetic Resonance 25 (2023): 40, 10.1186/s12968-023-00942-z. PMC10357639 37474977

[30] J. Schulz‐Menger , D. A. Bluemke , J. Bremerich , et al., “Standardized Image Interpretation and Post‐Processing in Cardiovascular Magnetic Resonance ‐ 2020 Update,” Journal of Cardiovascular Magnetic Resonance 22 (2020): 19, 10.1186/s12968-020-00610-6. PMC7066763 32160925

[31] K. Leong , L. Howard , F. L. Giudice , et al., “Utility of Cardiac Magnetic Resonance Feature Tracking Strain Assessment in Chronic Thromboembolic Pulmonary Hypertension for Prediction of REVEAL 2.0 High Risk Status,” Pulmonary Circulation 13 (2023): e12116, 10.1002/pul2.12116. PMC9947220 36843875

[32] S. Petersson , P. Dyverfeldt , and T. Ebbers , “Assessment of the Accuracy of MRI Wall Shear Stress Estimation Using Numerical Simulations,” Journal of Magnetic Resonance Imaging 36 (2012): 128–138, 10.1002/jmri.23610. 22336966

[33] S. Saitta , "saitta‐s/flow4D: flow4D. Zenodo" (2022), https://doiorg/10.5281/zenodo.7236015.

[34] G. Reiter , U. Reiter , G. Kovacs , H. Olschewski , and M. Fuchsjäger , “Blood Flow Vortices Along the Main Pulmonary Artery Measured With MR Imaging for Diagnosis of Pulmonary Hypertension,” Radiology 275 (2015): 71–79, 10.1148/radiol.14140849. 25372980

[35] T. H. Oechtering , A. Nowak , M. M. Sieren , et al., “Repeatability and Reproducibility of Various 4D Flow MRI Postprocessing Software Programs in a Multi‐Software and Multi‐Vendor Cross‐Over Comparison Study,” Journal of Cardiovascular Magnetic Resonance 25 (2023): 22, 10.1186/s12968-023-00921-4. PMC10052852 36978131

[36] C. Montalba , J. Urbina , J. Sotelo , et al., “Variability of 4D Flow Parameters When Subjected to Changes in MRI Acquisition Parameters Using a Realistic Thoracic Aortic Phantom,” Magnetic Resonance in Medicine 79 (2018): 1882–1892, 10.1002/mrm.26834. 28714282

[37] M. Cherry , Z. Khatir , A. Khan , and M. Bissell , “The Impact of 4D‐Flow MRI Spatial Resolution on Patient‐Specific CFD Simulations of the Thoracic Aorta,” Scientific Reports 12 (2022): 15128, 10.1038/s41598-022-19347-6. PMC9448751 36068322

