# Supplementary material for: 4D‐Flow MRI‐Derived Wall Shear Stress as a Non‐Invasive Biomarker for the Detection and Hemodynamic Characterization of Pulmonary Hypertension
Source: Pulm Circ. 2026 Oct 19;16(4):e70426. doi: 10.1002/pul2.70426 (PMC13678861; doi:10.1002/pul2.70426)
Supplement: Supplementary file 1 [file PUL2-16-e70426-s001.docx]

## Supplementary Files

### Table S1

| **Correlation Table: 4D-Flow WSS vs Functional CMR Measures**  Pearson’s correlation coefficient (r) | | | | |
| --- | --- | --- | --- | --- |
|  | **RVESVi** | **RVEF** | **RV Strain** | **RA Strain** |
| **Main Pulmonary Artery** |  |  |  |  |
| 3D WSS_peak_ | -0.62*** | 0.67*** | -0.50*** | 0.47** |
| 3D WSS_avg_ | -0.53*** | 0.56*** | -0.54*** | 0.38* |
| Axial 2D WSS_peak_ | -0.43** | 0.58*** | -0.41* | 0.53*** |
| Axial 2D WSS_avg_ | -0.38* | 0.53*** | -0.49*** | 0.40** |
| Circumferential 2D WSS_peak_ | -0.25 | 0.49*** | -0.40** | 0.38** |
| Circumferential 2D WSS_avg_ | -0.12 | 0.37* | -0.33* | 0.28 |
| **Right Pulmonary Artery** |  |  |  |  |
| 3D WSS_peak_ | -0.53*** | 0.54*** | -0.51*** | 0.22 |
| 3D WSS_avg_ | -0.48*** | 0.56*** | -0.59*** | 0.24 |
| **Left Pulmonary Artery** |  |  |  |  |
| 3D WSS_peak_ | -0.44** | 0.51*** | -0.45** | 0.23 |
| 3D WSS_avg_ | -0.48*** | 0.54*** | -0.53*** | 0.25 |
| **Global Wall Shear Stress** |  |  |  |  |
| 3D WSS_global_peak_ | -0.57*** | 0.62*** | -0.53*** | 0.33* |
| 3D WSS_global_avg_ | -0.54*** | 0.59*** | -0.61*** | 0.32* |
| **Caption**: Pearson’s correlation (r) of 4D flow measured wall shear stress with MRI measured functional parameters.  *Significance levels: * p < 0.05,** p < 0.01, *** p < 0.001* | | | | |

### Table S2

|  | **ROC Curve Analysis of WSS Measures to Predict PH** | | | | | | |
| --- | --- | --- | --- | --- | --- | --- | --- |
|  | | **Cut off**  **(pa)** | **AUC**  **(95%CI)** | **Sensitivity**  **(%)** | **Specificity**  **(%)** | **+ LR** | **-LR** |
| **Prediction of mPAP >20mmHg** | | | | |  |  |  |
| RPA WSS_peak_ | | 0.28 | 0.99  (0.98-1) | 93 | 96 | 21.3 | 0.08 |
| WSS_global_peak_ | | 0.30 | 0.98  (0.96-1) | 98 | 87 | 7.5 | 0.03 |
| WSS_global_avg_ | | 0.09 | 0.96  (0.92-1) | 86 | 96 | 19.6 | 0.15 |
| **Prediction of PVR>2 WU** | | | | |  |  |  |
| RPA WSS_peak_ | | 0.28 | 0.99  (0.98-1) | 93 | 100 | - | 0.07 |
| WSS_global_peak_ | | 0.30 | 0.99  (0.97-1) | 98 | 91 | 10.7 | 0.03 |
| WSS_global_avg_ | | 0.09 | 0.98  (0.96-1) | 86 | 100 | - | 0.14 |
| **Prediction of GLS <15.3** | | | | |  |  |  |
| LPA WSS_avg_ | | 0.08 | 0.88  (0.79-0.90) | 90 | 76 | 3.7 | 0.14 |
| WSS_global_peak_ | | 0.18 | 0.87  (0.77-0.97) | 79 | 87 | 5.9 | 0.24 |
| WSS_global_avg_ | | 0.09 | 0.87  (0.78-0.96 | 74 | 84 | 4.7 | 0.31 |
| **Prediction of RVSV/RVESV ration <1** | | | | |  |  |  |
| LPA WSS_avg_ | | 0.08 | 0.91  (0.83-0.97) | 82 | 86 | 5.9 | 0.21 |
| WSS_global_peak_ | | 0.18 | 0.93  (0.87-0.98) | 71 | 97 | 25.7 | 0.29 |
| WSS_global_avg_ | | 0.09 | 0.90  (0.83-0.96) | 90 | 78 | 4.0 | 0.14 |

**Caption**: ROC curve analysis of wall shear stress to detect pulmonary hypertension.

### Figure S1

**Figure S1**. Box Plots for comparison of Wall Shear Stress measures between subjects with and without pulmonary hypertension.

PH = pulmonary hypertension, LPA = left pulmonary artery, MPA = main pulmonary artery, RPA = right pulmonary artery, WSS = wall shear stress

### Figure S2

**Figure S2**. Box Plots for comparison of 3D Wall Shear Stress measures between patients with chronic thromboembolic pulmonary hypertension and those with pulmonary arterial hypertension.

CTEPH = chronic thromboembolic pulmonary, PAH = pulmonary arterial hypertension, LPA = left pulmonary artery, MPA = main pulmonary artery, RPA = right pulmonary artery, WSS = wall shear stress
